# Diagnostic Concordance of Telemedicine as Compared With Face-to-Face Care in Primary Health Care Clinics in Rural India: Randomized Crossover Trial

**DOI:** 10.2196/42775

**Published:** 2023-06-23

**Authors:** Neha Verma, Bimal Buch, Radha Taralekar, Soumyadipta Acharya

**Affiliations:** 1 Intelehealth Baltimore, MD United States; 2 Center for Bioengineering Innovation & Design Johns Hopkins University Baltimore, MD United States

**Keywords:** telemedicine, telehealth, eHealth, opensource, digital assistant, diagnostic concordance, COVID-19, primary care, rural health, teleconsultation, patient care

## Abstract

**Background:**

With the COVID-19 pandemic, there was an increase and scaling up of provider-to-provider telemedicine programs that connect frontline health providers such as nurses and community health workers at primary care clinics with remote doctors at tertiary facilities to facilitate consultations for rural patients. Considering this new trend of increasing use of telemedicine, this study was conducted to generate evidence for patients, health providers, and policymakers to compare if provider-to-provider telemedicine-based care is equivalent to in-person care and is safe and acceptable in terms of diagnostic and treatment standards.

**Objective:**

This study aims to compare the diagnosis and treatment decisions from teleconsultations to those of in-person care in teleclinics in rural Gujarat.

**Methods:**

We conducted a diagnostic concordance study using a randomized crossover study design with 104 patients at 10 telemedicine primary care clinics. Patients reporting to 10 telemedicine primary care clinics were randomly assigned to first receive an in-person doctor consultation (59/104, 56.7%) or to first receive a health worker–assisted telemedicine consultation (45/104, 43.3%). The 2 groups were then switched, with the first group undergoing a telemedicine consultation following the in-person consultation and the second group receiving an in-person consultation after the teleconsultation. The in-person doctor and remote doctor were blinded to the diagnosis and management plan of the other. The diagnosis and treatment plan of in-person doctors was considered the gold standard.

**Results:**

We enrolled 104 patients reporting a range of primary health care issues into the study. We observed 74% (77/104) diagnostic concordance and 79.8% (83/104) concordance in the treatment plan between the in-person and remote doctors. No significant association was found between the diagnostic and treatment concordance and the order of the consultation (*P*=.65 and *P*=.81, respectively), the frontline health worker–doctor pair (both *P*=.93), the gender of the patient (both *P*>.99), or the mode of teleconsultation (synchronous vs asynchronous; *P*=.32 and *P*=.29, respectively), as evaluated using Fisher exact tests. A significant association was seen between the diagnostic and treatment concordance and the type of case (*P*=.004 and *P*=.03, respectively). The highest diagnostic concordance was seen in the management of hypertension (20/21, 95% concordance; Cohen kappa=0.93) and diabetes (14/15, 93% concordance; Cohen kappa=0.89). The lowest values were seen in cardiology (1/3, 33%) and patients presenting with nonspecific symptoms (3/10, 30%). The use of a digital assistant to facilitate the consultation resulted in increased adherence to evidence-based care protocols.

**Conclusions:**

The findings reflect that telemedicine can be a safe and acceptable alternative mode of care especially in remote rural settings when in-person care is not accessible. Telemedicine has advantages. for the potential gains for improved health care–seeking behavior for patients, reduced costs for the patient, and improved health system efficiency by reducing overcrowding at tertiary health facilities.

## Introduction

Equitable access to primary health care remains an elusive dream in rural India [1]. The rural health system faces a shortage of health workers at every level [2]. Almost 77% of all qualified health workers in India are based in urban centers where 31% of the population resides [2]. Nurses, community health workers, and other paramedical personnel at the primary health care level can manage some part of the disease burden. However, several commonly seen primary health conditions such as management of hypertension, diabetes, family planning, severe malnutrition, high-risk pregnancies, dermatological conditions, and osteoarthritis require intervention from a doctor, and such patients are referred to higher-level health centers such as a primary health center (PHC) or a community health center (CHC). The average distance traveled to a PHC is 8.7 km or to a CHC is 17.64 km [3], making physical visits to a doctor challenging to access for remote areas, especially where transportation is a challenge.

One emerging model of mitigating the primary health care access gap is provider-to-provider telemedicine (TM) [4]. This provider-to-provider TM approach has been envisioned in India’s Ayushman Bharat (AB) program to connect paramedical frontline health providers at 150,000 health and wellness centers (HWCs) to doctors at secondary or tertiary facilities [5,6]. The Indian Government’s eSanjeevani AB-HWC TM system that operates in a hub-and-spoke model facilitating consultations between HWCs (spokes) and tertiary hospitals (hubs) [6] has already achieved over 6.7 million consultations [7]. With over 2000 hubs [7] and 28,000 spokes [7], the program demonstrates that this approach is feasible and expands health access, especially in the context of the COVID-19 pandemic. This approach can bridge the urban-rural disparity in the availability of trained medical professionals, improve the quality of available primary health services, and reduce patient referrals [8]. However, an important consideration for patients, providers, and policymakers is whether virtual doctors’ visits facilitated by a frontline health worker are an appropriate standard of care in this setting and an acceptable alternative when in-person doctor visits are not possible.

The authors of this paper developed an enhanced provider-to-provider TM approach in which a frontline health worker empowered by an appropriate job aid or training can meaningfully interact with the patient to elicit clinical signs and symptoms and share them with the help of a synchronous or asynchronous network of TM doctors for a diagnosis and management plan [9]. We studied the diagnostic concordance and provider acceptability of this provider-to-provider TM program that connected nurses in primary health care facilities with remote teledoctors through a randomized crossover study in 10 rural HWCs in the Morbi district of Gujarat.

Several TM studies in developed countries have shown that TM can provide comparable care at lower costs and greater convenience for patients [10,11]. Another study reinforced that TM can be a great opportunity in the care of disadvantaged populations and has the potential to break down inequalities [12]. TM is an important tool for health care delivery and has achieved even greater significance in the context of the COVID-19 pandemic [13,14]. However, there are certain limitations of TM-based care. There are few rigorous systematic evaluations on the clinical benefits of TM and the impact of TM on the quality and safety of care [15]. Therefore, it is essential to understand when TM can deliver comparable diagnostic outcomes and whether it provides high-quality care.

Various studies have shown good or very good levels of diagnostic concordance between face-to-face (F2F) and TM-based care with concordance rates between 73% and 99% [16-22]. However, very few studies have been conducted comparing teleconsultation's diagnostic and treatment outcomes to those of F2F consultations in developing countries. We found only 1 such study that showed 78% diagnostic concordance and 89% treatment concordance between an in-person nurse visit and a virtual nurse visit for an asynchronous TM program in Kenya [18]. The impact of TM in developing countries is greater where there are significant geographic disparities in the distribution of available health care professionals, especially between urban and rural areas [4]. However, there is a gap in the literature with very few randomized evaluations of TM. Systematic randomized evaluations of TM are possible but rarely undertaken or published in developing countries due to financial and resource constraints [18].

In addition, our TM program used a task-shifting digital assistant, called “Ayu,” that provided just-in-time job aids to nurses so they could collect a comprehensive patient history and physical exam information to share with the remote physician [9]. A major concern in the use of this digital assistant is its impact on the overall quality of the consultation. If the nurse collects too little or incorrect information, it could adversely impact the quality of the consult. At the very least, the digital assistant should maintain diagnostic accuracy in a consult and increase the overall efficiency of the consult. In this study, we also examined the impact of this digital assistant on the overall consult quality and efficiency.

## Methods

### Study Site

**Figure 1:**
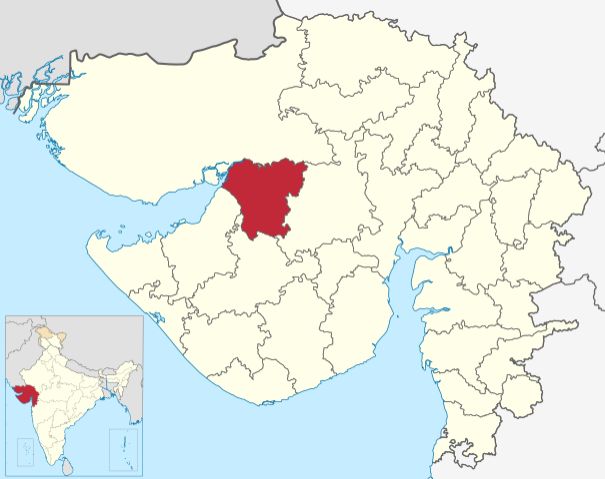
Location of Morbi district. (reproduced from Milenioscuro [23], which is published under Creative Commons Attribution 4.0 International License [24]).

The MyTeleDoc project is a TM network in which community health officers (CHOs) at HWCs can facilitate virtual visits with doctors using a mobile app. CHOs use the app for patients presenting with health conditions that they cannot manage and otherwise refer to a higher-level facility. The project's pilot phase was implemented at 22 HWCs in 2 blocks of Morbi district—Maliya and Tankara (see district location in Figure 1; ie, in 11% [22/198] of the total HWCs in the district). We randomly selected 10 HWCs of the total 22 HWCs in the project for this study. The Morbi District Administration implemented the pilot project in HWCs in difficult-to-reach parts of the district. Thus, these HWCs served a patient population with poorer health and socioeconomic indicators than the district median and the median for Gujarat.

These HWCs have outpatient primary care clinics run by the CHOs [5] who are trained nurses or Ayurveda practitioners with additional training in comprehensive primary health care delivery received through an accredited bridge training program [25]. They also conduct screening programs and community health activities. Using primary data collected from the district health administration, we found a sufficient number of general practitioners within the Morbi government health system. However, the district faces a severe shortage in the number of available specialists, with only 1 internal medicine physician, 1 pediatrician, and no obstetricians nor gynecologists in the district health administration for the entire population of 10 lakh (1 million) people. This dearth of specialist providers in the public health system, and especially of obstetricians and pediatricians [26], is also seen all over Gujarat, with 97% of posts vacant, as per 1 study [25]. The project recruited general practitioners (called medical officers) from within the Morbi health system and volunteer specialists from private facilities and nongovernmental organizations in the nearby district of Rajkot to serve as remote doctors for the project. These were allopathic doctors with an MBBS or MD degree. Thus, the project aimed to reduce health access barriers for rural patients while also increasing the available capacity of providers in the district through private sector support. This project was implemented just before the COVID-19 pandemic in December 2019 and was used during the pandemic to remotely manage patients and reduce referrals to crowded tertiary facilities overburdened with COVID-19 patients.

### Sample Size

Our study sample consisted of 104 patients attending 10 randomly selected TM primary care clinics in Morbi district. These patients were randomly assigned to first receive an in-person doctor consultation (59/104, 56.7%) or to first receive a health worker–assisted TM consultation (45/104, 43.3%). The 2 groups were then switched, with the first group undergoing a TM consultation following the in-person consultation and the second group receiving an in-person consultation after the teleconsultation.

### Technology

**Figure 2:**
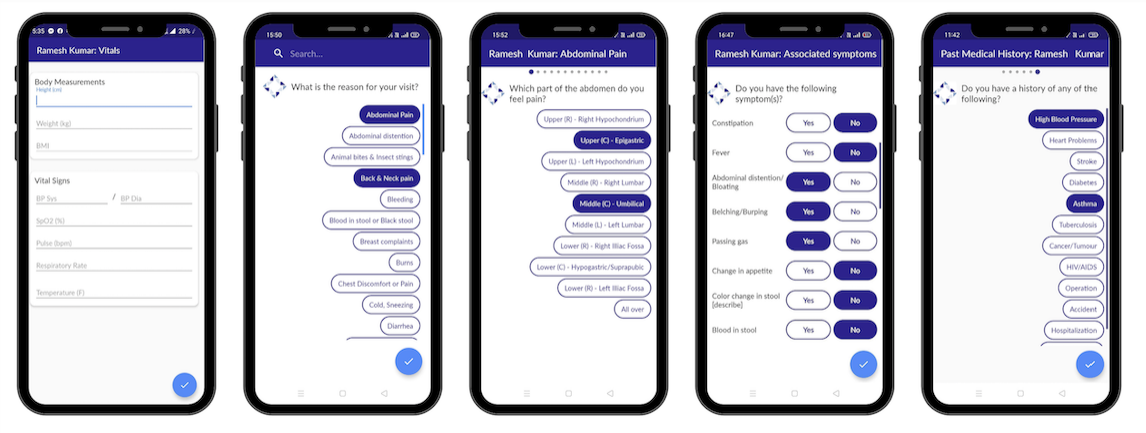
MyTeleDoc application with the digital assistant Ayu.

**Figure 3:**
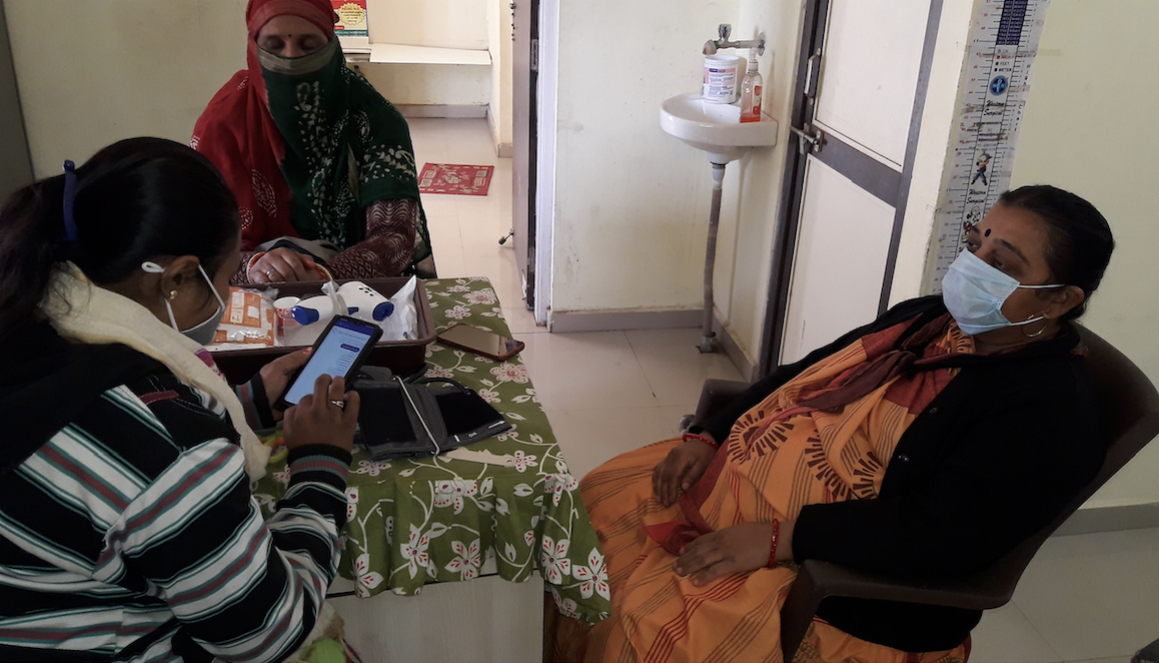
A community health officer using the digital assistant Ayu in the MyTeleDoc app to collect a patient history and facilitate a teleconsultation.

The MyTeleDoc app is a white-labeled version of an open-source TM software product called Intelehealth [9]. It is distributed as a digital public good and is free to adapt, use, and implement by health organizations [9]. A description of the platform's design, development, and architecture is provided in a prior publication [9]. The MyTeleDoc app consists of a digital health assistant called Ayu that guides the CHO in collecting a comprehensive patient history through adaptive questionnaires depending on the patient’s presenting complaint. The CHO uploads the case to a remote doctor who views it through a mobile app for doctors. The doctor conducts a video or audio call with the CHO or the patient and provides an e-Prescription with a diagnosis, medicines, tests, advice, or referral (synchronous teleconsultation). At the doctor's discretion, they may also provide a diagnosis and triage decision without speaking with the CHO or the patient if they feel the information from the CHO is sufficient for them to arrive at a management plan for the patient (asynchronous teleconsultation). The CHO can then discuss the diagnosis with the patient, dispense the required medications, and manage follow-up care as needed. We trained CHOs to use the app and TM-based care over 1.5 days of training. Figures 2 and 3 show the MyTeleDoc CHO app and digital assistant.

### Ethical Approval

Ethical approval for the study was obtained from a local ethics committee in India from the DY Patil Institute of Medical Sciences and the Johns Hopkins University Institutional Review Board (IRB0208185). We enrolled patients from all age groups in need of curative primary health care services but not emergent care at the 10 HWCs that participated in the study. CHWs called Accredited Social Health Activist (ASHA) workers identified patients meeting the study's inclusion and exclusion criteria and contacted them through home visits inviting them to participate in the study. In addition, the ASHA and CHO also approached patients who came to the HWC on the study day to participate. Several patients in the Morbi district were screened during hypertension and diabetes screening camps; they were also invited to participate in the study to confirm their diagnosis and adjust medications as needed. All patients who indicated an interest in participating underwent a consent process through an IRB-approved consent form conducted in the local language of Gujarati. A study team member verbally read the consent form developed in simple language and available in Gujarati explaining the study procedures, risks, and limitations. Adequate time was given for patients to understand the study and ask questions. Since the study involved a randomized crossover design and patients would need to spend more than twice as much time at the clinic as they usually would, patients were compensated ₹500 (US $6.12) for participation. CHOs were compensated ₹2500 (US $30.59) for their participation in the study. The 11 ASHAs who participated in patient enrollment were compensated ₹500 (US $6.12) for the same. The participating doctors received no additional compensation for this study.

### Study Protocol

After enrollment, we randomly allocated one-half of the patients to receive a F2F doctor consultation (standard of care). This was followed by a CHO-facilitated teleconsultation with a remote doctor. The other one-half were randomly allocated to receive the teleconsultation first, followed by the F2F consultation (randomized crossover design) to control for order and crossover effects. A gap of 30 minutes to 90 minutes was given between the consultations. Doctors participating in the study were highly experienced family physicians with more than 30 years of work experience to minimize misdiagnosis due to provider skill level. Both consultations occurred in separate rooms, and participating providers in each arm were blinded to the outcomes of the other. Patients were instructed to share the same health issue during both the consultations and not discuss the results of one consultation with the other doctor to minimize the effect of one treatment arm biasing the other. Participating patients received the standard of care (ie, the in-person consultation treatment plan).

The in-person doctor used a paper form to record the patient's presenting complaints, physical exams, medications, and treatment plan during the F2F consultation. During the teleconsultation, the CHO used the MyTeleDoc app to elicit and document the patient's history and physical exam findings and share the same with the remote doctor, who then used the MyTeleDoc doctor's app to share the medications and treatment plan. All patients were assigned a unique identifier. After the study concluded, a study team member entered the data from the F2F consultation from the paper forms into a digital format. We extracted the data from the teleconsultation from the MyTeleDoc portal. We excluded personal identifiers such as name, phone number, address, and face photographs from the F2F consultation and teleconsultation records. We included age, gender, occupation, and physical exam images of the patient's body in the data extraction due to their relevance to the treatment plan. We matched the F2F and teleconsultation patient records using the patient identifier. Similarly, we did not include information that would identify the provider, such as the name of the CHO and doctor, and replaced them with a pseudo-identifier. However, due to the small number of CHOs (n=10) and doctors (n=3), complete anonymization was not possible.

We compared the medical records from the teleconsultation and F2F consultation to determine the concordance between the 2 modalities. For our study, we considered the F2F diagnosis as the gold standard. We measured the concordance as the percentage of cases for which the F2F and TM physician had the same diagnosis (diagnostic concordance) and treatment (treatment concordance). Percent agreement has been used to measure interrater agreement in several similar nonrandomized studies on TM [18,19].

The doctors participating in the study discussed the patient cases to determine if the in-person care and TM-based care provided were substantially similar in diagnosis and treatment plan. Allowable differences in treatment planning, such as medication prescribing preferences or differences in how diagnoses were entered, were not considered discordant. While analyzing the impact of the digital assistant on the teleconsultation outcome, the doctors discussed the cases and put in descriptive notes for each case. To minimize bias, the final analysis was audited by another study team member (NV) who was not one of the participating doctors. Changes were made in 1 record after the audit, during which we found a data entry error, and the diagnostic concordance for that case was changed from concordant to discordant. The descriptive notes were analyzed and summarized by a study team member (NV) who also conducted the data analysis using SPSS (IBM Corp) and Excel (Microsoft).

## Results

### Sample

We selected 10 HWCs and enrolled 105 patients across all the study locations. We excluded 1 patient case, as the paper form for the F2F encounter was misplaced, bringing a total of 104 patients into the study. Table 1 shows the gender and age (self-reported) distribution of patients.

**Table 1:** Age and gender distribution of patients (n=104).

Variables			Results, n (%)
**Gender**			
	Female	70 (67.3)	
	Male	34 (32.7)	
**Age group (years)**			
	0-5	12 (11.5)	
	6-18	5 (4.8)	
	19-35	18 (17.3)	
	36-60	36 (34.6)	
	61-100	33 (31.7)	

A total of 10 CHOs participated in the study, 1 at each HWC. The average age of CHOs was 27 years. All the CHOs had a bachelor’s degree in nursing. The total number of years of work experience as a nursing professional before becoming a CHO varied from 1 year to 6 years. They were very comfortable using technology and had undergone training to use the app. We enrolled 3 doctors in the study, 2 to provide in-person care and 1 for remote care. The average age of these physicians was 63 years, and each had over 30 years of experience as a family physician. The physicians routinely used smartphones and were comfortable using the TM portal. All providers were fluent in Gujarati, Hindi, and English.

### Diagnostic and Treatment Concordance Between F2F and TM Consultations

Overall, we observed 74% (77/104) diagnostic concordance and 79.8% (83/104) treatment concordance between F2F and TM consultations. There was no statistically significant difference in the diagnostic concordance (*P*=.65) or the treatment concordance (*P*=.81), as assessed using 2-sided Fisher exact tests, between the cases where F2F or TM consultation was conducted first. Similarly, we could not find any statistically significant association between the diagnostic or treatment concordance and other key variables that may influence the consultation, such as the CHO-doctor pair, gender of the patient, and mode of consultation (see Table 2 for *P* values).

**Table 2:** Diagnostic and treatment concordance with patient and visit characteristics.

Variables				Patients, n (%)		Diagnostic concordance, n (% agreement)		2-sided *P* value^a^		Treatment concordance, n (% agreement)		2-sided *P* value^a^
**Order of consultation**												
		Overall	104 (100)		77 (74)		.65		83 (80)		.81	
	F2F^b^ consultation first		59 (56.7)		45 (76.2)				48 (81.3)			
	TM^c^ consultation first		45 (43.2)		32 (71.1)				35 (77.7)			
**Location/CHO^d^-doctor pair**												
		Overall	104 (100)		77 (74)		.93		83 (80)		.93	
	HWC^e^ 1		11 (11)		8 (72.7)				10 (90.9)			
	HWC 2		11 (11)		8 (72.7)				9 (81.8)			
	HWC 3		8 (8)		4 (50)				5 (62.5)			
	HWC 4		10 (10)		7 (70)				8 (80)			
	HWC 5		10 (10)		7 (70)				7 (70)			
	HWC 6		10 (10)		8 (80)				8 (80)			
	HWC 7		13 (13)		11 (84.6)				11 (84.6)			
	HWC 8		9 (9)		7 (77.7)				8 (88.8)			
	HWC 9		11 (11)		9 (81.8)				9 (81.8)			
	HWC 10		11 (11)		8 (72.7)				8 (72.7)			
**Gender**												
	Overall		104 (100)		77 (74)		>.99		83 (79.8)		*>*.99	
	Female		70 (67.3)		52 (74.2)				56 (80)			
	Male		34 (32.6)		25 (73.5)				27 (79.4)			
**Type of case/specialty**												
	Overall		113 (100)		84 (74.3)		.004		90 (79.6)		.03	
	Hypertension		21 (18.5)		20 (95.2)				20 (95.2)			
	Diabetes		15 (13.2)		14 (93.3)				14 (93.3)			
	Obstetrics		10 (8.8)		8 (80)				8 (80)			
	Pediatrics		17 (15)		13 (76.4)				15 (88.2)			
	Orthopedics		18 (15.9)		13 (72.2)				14 (77.7)			
	Gastroenterology		6 (5.3)		4 (66.6)				4 (66.6)			
	Dermatology		8 (7.1)		5 (62.5)				6 (75)			
	Gynecology		5 (4.4)		3 (60)				3 (60)			
	Cardiology		3 (2.7)		1 (33.3)				1 (33.3)			
	Miscellaneous		10 (8.8)		3 (30)				5 (50)			
**Mode of teleconsultation**												
	Overall		100 (100)		73 (73)		.32		79 (79)		.29	
	Asynchronous		84 (84)		60 (71.4)				65 (77.3)			
	Synchronous		16 (16)		13 (81.2)				14 (87.5)			

^a^Fisher exact tests were used to determine if there was a significant association between diagnostic and treatment concordance and the order of consultation, CHO-doctor pair, gender, type of case and mode of teleconsultation.

^b^F2F: face-to-face.

^c^TM: telemedicine.

^d^CHO: community health officer.

^e^HWC: health and wellness center.

Since a patient may have multiple diagnoses, a patient case could be classified into multiple specialties. We observed statistically significant associations between the type of case and the diagnostic concordance (*P*=.004) and treatment concordance (*P*=.03), as assessed using 2-sided Fisher exact tests*.*

### Interrater Reliability for Diagnosis of Hypertension and Diabetes (Cohen Kappa)

Participants included 21 patients with diabetes and 17 patients with hypertension. Historic blood pressure and random blood glucose values were available from the nurse for these patients. Thus, the diagnoses of hypertension and diabetes were based on multiple readings taken in the clinic over several visits. Due to the wide range in diagnoses, Cohen kappa, which is accepted as a more robust measure of concordance [27,28], could only be calculated for the most common diagnoses for which there was a sufficient sample size—hypertension and diabetes. The F2F physician diagnosed 21 patients as hypertensive. The sensitivity of a hypertension diagnosis in the TM encounter compared with a F2F consultation was 0.95, and the specificity was 0.97. The Cohen kappa for a hypertension diagnosis was 0.89, indicating strong agreement. An in-person physician diagnosed 15 patients with type 2 diabetes. The sensitivity of a diabetes diagnosis in the TM encounter compared with a F2F consultation was 0.93, and the specificity was 0.99. The Cohen kappa for a diabetes diagnosis was 0.93, indicating near-perfect agreement. Since this was a diagnostic setting, the TM physician gave a higher focus on specificity.

### Impact of the Digital Assistant on the Teleconsultation Outcome

The digital assistant allowed the CHO to collect a lot of the initial clinical information and share it with the doctor, and in 84% of the cases, the doctor felt this information was sufficient to arrive at a diagnosis and did not feel the need to speak with the patient. This allowed most consultations to be delivered asynchronously. The output clinical notes also allowed the remote doctor to spend less time on the diagnosis and resulted in a more efficient use of their time. CHOs reported that they were able to assess a patient thoroughly and were less likely to skip any steps. Finally, it eliminated the documentation burden for the remote doctor. We observed that the skill of the CHO in using the digital assistant was very important. In at least 12 of the 27 (44%) discordant cases, missing, incorrect, or incomplete information provided by the CHO led to a discordant diagnosis.

### Limitations of TM

The majority of patients (60/104, 57.7%) had just 1 diagnosis; 30.8% (32/104) of patients had 2 diagnoses, 10.6% (11/104) of patients had 3 diagnoses, and 1% (1/104) of patients had 4 diagnoses. Across all 104 patients, there were a total of 65 diagnoses and 162 patient-diagnosis pairs (see Table 3).

**Table 3:** List of diagnoses (104 patients with 162 patient-diagnosis pairs).

Number	Diagnosis	Patients, n
1	Essential hypertension	29
2	Diabetes mellitus, type 2	17
3	Anemia	11
4	Osteoarthritis	8
5	Routine ANC^a^ checkup	6
6	Anxiety neurosis	5
7	Sciatic neuralgia	4
8	Atopic dermatitis	4
9	Soft tissue injury	3
10	Rheumatoid arthritis	3
11	Malnourished child	3
12	Helminthiasis	3
13	Vitamin A deficiency	2
14	Vaginal discharge syndrome	2
15	Upper respiratory tract infection	2
16	Ringworm	2
17	Psoriasis	2
18	Polyneuritis	2
19	Myositis around the shoulder	2
20	Myalgia	2
21	High-risk ANC checkup	2
22	General debility	2
23	Gastritis	2
24	Constipation	2
25	Cataract	2
26	Vitamin C deficiency	1
27	Viral fever	1
28	Ventral hernia	1
29	Urinary tract infection	1
30	Tinea versicolor	1
31	Scabies	1
32	Sacroiliac strain	1
33	Rule out angina pectoris	1
34	Residual hemiplegia	1
35	Residual hemiparesis	1
36	Recurrent tonsilitis	1
37	Pruritus	1
38	Primary sterility	1
39	Preeclampsia	1
40	Postural hypotension	1
41	Post-ringworm hyperpigmentation	1
42	Post-pimple scarring	1
43	Peripheral neuritis	1
44	Perimenopausal syndrome	1
45	Nonhealing ulcer	1
46	Ischemic heart disease	1
47	Intrauterine growth restriction	1
48	Infected psoriasis	1
49	Hyperthyroidism	1
50	High arched feet deformity	1
51	Hemorrhoids	1
52	Gall stones	1
53	Exertional dyspnea	1
54	Esophageal cancer	1
55	Early cardiac failure	1
56	Congenital valvular heart disease	1
57	Chronic kidney disease	1
58	Cervical spondylosis	1
59	Cerebral palsy	1
60	Calcium deficiency	1
61	Bronchopneumonia	1
62	Atheromatous arthritis	1
63	Ankle sprain	1
64	Allergic dermatitis	1
65	Age-related debility	1

^a^ANC: antenatal care.

Of these, 113 diagnoses were considered “primary,” for which the diagnosis corresponded with the patient's presenting complaints. The remaining 49 diagnoses were considered “secondary” or incidental findings, for which the patient was not complaining specifically of this health issue, but the in-person physician identified these during a routine physical exam. The remote physician did not identify these in the TM encounter due to their inability to conduct a physical exam. The CHO would also likely not have the proficiency to conduct a high-quality exam and identify these salient findings that an experienced clinician observed.

Of note, the missed diagnoses included anemia, cataract, osteoarthritis, anxiety disorder, and a potential esophageal cancer. Physical examination and careful history taking are essential to diagnosing these conditions. We observed that, although TM can help identify and treat the root cause of a patient's primary concerns, diseases without any prominent presentation or symptoms negatively impacting the patient's quality of life would likely be missed and can be identified only during a routine review of systems. However, this observation may be due to the nature of our study, in which the in-person doctor provided a thorough consultation, spending more than 20 minutes per patient. In a busy outpatient setting, the physician may not have the time to conduct a review of systems and would likely only address the patient's presenting complaint. In addition, we observed that many study participants had multiple unaddressed comorbidities, which we attributed to the health access gap resulting in patients' delay in seeking primary health care, an underlying issue that TM solves.

## Discussion

### Principal Findings

The results of our study suggest that TM-based care is comparable with in-person care with acceptable concordance between F2F and TM-based diagnosis and treatment of primary care conditions. TM is a viable and acceptable alternative when in-person care is challenging to access. We observed that rural patients would likely ignore their health issues due to geographic and financial access barriers; however, the availability of TM at primary health care centers through digital assistance with a CHO would ease these challenges. TM provides equitable access and availability of health care services without compromising the acceptable standards of care as reflected in this study, with 74% and 80% diagnostic and treatment concordance between F2F and TM-based services.

It is important to remember that this study measured concordance in diagnosis between the 2 physicians and not diagnostic accuracy since a reference standard was unavailable. Physicians often disagree on patients' diagnoses and management plans, even during in-person consultations. A study by the Mayo Clinic of the diagnostic agreement between a first consultation and second opinion found that the 2 providers completely agreed in 12% of cases, the diagnosis was further refined by the second physician in 66% of cases, and a completely different diagnosis was provided in 21% of cases [29]. A teledermatology study had 174 patients first examined by 2 in-person dermatologists, followed by the patient images and records being analyzed by 2 teledermatologists [20]. The agreement between the in-person dermatologists was 83.3%, and the agreement between the teledermatologists was 81% [20]. The agreement between in-person and teledermatologists was between 78.2% and 83.9% [20]. We identified several studies that reported diagnostic concordance between TM and F2F care and summarized these in Table 4. All the studies referenced in Table 4 concluded that TM was an acceptable alternative to in-person care.

**Table 4:** Results of studies reporting diagnostic concordance in telemedicine versus face-to-face (F2F) care.

Number	Author, year	Title	Sample size	Concordance measure	Diagnostic concordance	Treatment concordance
1	Smith et al, 2008 [16]	Concordance between real-time telemedicine assessments and face‐to-face consultations in paediatric otolaryngology	68	Percent agreement	99%	93%
2	Edison et al, 2008 [17]	Diagnosis, diagnostic confidence, and management concordance in live-interactive and store-and-forward teledermatology compared to in-person examination	110	Percent agreement	80% (F2F vs synchronous teleconsultation); 73% (F2F vs asynchronous teleconsultation)	—^a^
3	Qin et al, 2012 [18]	Reliability of a telemedicine system designed for rural Kenya	102	Percent agreement	78.4%	89.2%
4	Seim et al, 2017 [19]	Developing a synchronous otolaryngology telemedicine clinic: prospective study to assess fidelity and diagnostic concordance	21	Percent agreement	95%	—
5	Keller et al, 2020 [20]	Inpatient teledermatology: diagnostic and therapeutic concordance among a hospitalist, dermatologist, and teledermatologist using store-and-forward teledermatology	100	Percent agreement	84.9% (cases with partial agreement); 52.8% (cases with complete agreement)	—
6	Fonseca et al, 2016 [21]	Validation of videoconference with smartphones in telemedicine facial trauma care	50	Kappa statistic	kappa=0.720 (physical exam findings); kappa=0.899 (computed tomography findings)	kappa=0.891
7	Gacto-Sánchez et al, 2020 [22]	Diagnostic accuracy of a telemedicine tool for acute burns diagnosis	202	Kappa statistic	kappa=0.94	—

^a^Not reported in the article.

In the context of these data, a concordance of 74% between an in-person and remote physician seems to be similar but marginally lower than the concordance seen between any 2 in-person physicians in other concordance studies of TM versus F2F care. However, it must be noted that, except for the study conducted in Kenya [18], all the other diagnostic concordance studies were conducted in developed countries where the doctor spends a lot more time with the patient and where diagnostic accuracy is known to be high compared with resource-limited settings where provider skill and time constraints impact the overall quality of the consult. Thus, we can conclude that the use of the digital assistant at the very least does not adversely impact the quality of the consult and that a nurse and remote doctor care team can use it reliably to achieve comparable diagnostic and treatment outcomes. However, we cannot say for sure what gains the assistant can bring about in improving the overall quality of the diagnosis, and this must be a topic of future studies.

We can conclude that the digital assistant enables adherence to evidence-based care delivery. A study by Das and Hammer [30] in Delhi showed that, for children presenting with diarrhea, only 25% of providers asked about blood or mucous in the stool, 49% asked whether the child had a fever, and 7% checked for a depression in the skull fontanel. Ayu prompts a nurse to capture all this information. The same study also showed that, on average, a primary care consultation lasts 3 minutes and patients are asked 3 questions, undergo 1 physical exam, and are given 3 different medications [30]. More than one-third of consultations last less than 1 minute, with 1 question being asked and no examinations conducted [30]. When compared with these metrics, Ayu allows for a higher standard of care to be provided. On average, a CHO spent 7 minutes taking a patient history and physical exam, with the doctor spending an additional 5 minutes. At least 7 to 10 questions were asked of every patient, and 9 physical exams were conducted including capturing the patient’s vital signs. Additionally, all the patient information captured by the CHO in the digital records was considered sufficient by 84% of the doctors and assisted them to proceed directly to other areas of examination.

The prevalence of hypertension in rural India is estimated to be 27.6% [31], and the prevalence of type 2 diabetes in rural India is 16% [32], significantly contributing to the burden of disease. TM was highly effective in the remote diagnosis and management of hypertension and diabetes. At the point of care, the nurse can complete the necessary physical examinations, such as collecting multiple blood pressure readings and measuring random blood glucose. CHOs routinely conduct screening camps for hypertension and diabetes, and patients identified as high risk are referred to a PHC for diagnosis and treatment by a general physician, as CHOs are not trained to diagnose nor initiate treatment for hypertension or diabetes. However, compliance with this referral was reported as poor by CHOs. Medications for the treatment of hypertension and diabetes are freely available at HWCs under the AB program and are reliably dispensed by CHOs. Thus, using TM to provide an initial diagnosis and continuous follow-up care for patients with chronic diseases reduces referrals and potential loss to follow-up. This is an interesting application area for TM, especially in a rural primary care center, because CHOs experience challenges accessing available doctors.

Our study also indicates that TM may be a feasible option for remotely providing antenatal care (ANC) during the COVID-19 pandemic, with 80% diagnosis concordance seen in care management. Several CHOs reported that pregnant women missed ANC visits during the pandemic, and patients were wary of referrals due to the fear of infection at a facility. However, a concerning case of intrauterine growth restriction and preeclampsia was incorrectly diagnosed in the TM encounter, indicating that teledoctors should be cautious during remote management of high-risk pregnancies. Our study also indicated that pediatric outpatient care might be a suitable use case for TM. These use cases are beneficial in a resource-limited setting like Morbi with no obstetrician or gynecologist and just 1 pediatrician on staff for the entire district. However, the small sample size of patients makes it difficult to conclude, and further research is required.

We observed that the diagnostic concordance for musculoskeletal issues was lower, at 72%, primarily due to the inability to conduct a physical examination. Reliability in teledermatology was poor, at 63% interrater agreement. However, this may be because the participating physicians in the study were not dermatologists. There were very few cardiology cases in the study population (n=3), of which only 1 had a concordant diagnosis. The remote physicians' inability to perform an exam for postural hypotension and conduct auscultation likely contributed to the lower diagnostic concordance. The physicians participating in the study also noted that patients presenting with nonspecific symptoms like dizziness, lethargy, or general body ache were challenging to diagnose, and this would have been the case even in an in-person setting. Hence, the patients categorized as “Miscellaneous” had an inferior interrater agreement (30%).

Several important factors can be addressed during TM encounters to improve their quality. Overall, synchronous teleconsultations were more reliable than asynchronous (81% vs 71% diagnostic concordance), though the difference was not statistically significant *(P*=.32). Additional training can be provided to CHOs to better capture the patient history. Improved usability of the digital assistant interface to minimize user error could also improve the consultation quality. Additional training can be given to doctors to better communicate with the CHO and patient. Creation of clinical guidelines by the Indian government around the effective use of TM would also help improve doctors’ ability to identify which patients can be treated remotely and who needs to be referred to in-person care.

Overall, although we observed tradeoffs in TM, compared with the benefits for rural low-income populations in improving health access, these are acceptable. Several studies have shown high levels of patient satisfaction with TM [18,33-36] and noted that TM results in significant cost savings for patients in rural communities [37-40].

Though TM is a new digital platform in rural areas for providing health care services and has multiple pros and cons, its value cannot be underestimated, as evident by our study and many other studies emulating the larger possibilities and implications. Our study is providing a small glimpse of TM service delivery to be explored through the involvement of paramedical workers like CHOs. This provision enables doctors to have more quality time to examine and evaluate the patient clinically. More studies may be needed to evaluate the long-term acceptability of TM rather than a 1-time consultation from the patients’ perspectives and its role in replacing F2F consultation wherever doctors are not available for equitable health care.

### Limitations of This Study

This study measured concordance, not accuracy, since the study intended to understand how TM compared with the standard of care. Because 2 providers can always disagree on the diagnosis, we cannot comment on the accuracy of diagnosis in either treatment arm. Observer bias was evident; the in-person doctor and the CHO and remote doctor pair took more time than they usually would to assess the patient. However, this was likely equal in both arms of the study.

Although patients were instructed during the consent process to share the same presenting complaint and discuss the same illness in both consultations, we did observe a few patients sharing different issues with the in-person doctor and the CHO-remote doctor care team. This might be due to the perception that they have already seen 1 provider, so now they should discuss a different issue with the other provider. It may also be because patients may view the CHO as less experienced and may discuss more basic health issues with the CHO, reserving the more complex issue for the doctor. Examining the medical records, we estimated that 6.7% (7/104) of patients may have shared different complaints in the consultations, of which 3 cases had a concordant diagnosis and 4 had a discordant diagnosis. However, this effect appears to be minimal, and the actual diagnostic agreement may be slightly higher. A study in Kenya referenced earlier in this paper also reported similar issues [18].

### Conclusion

Provider-to-provider TM, in which nurses at HWCs connect with remote physicians to provide care for patients they would otherwise have referred to a tertiary facility, is a feasible and safe intervention. TM is less reliable than in-person care but a safe and effective alternative where in-person care cannot be provided. We found it most effective for managing high-burden primary health conditions such as hypertension, diabetes, and remote ANC. The use of a digital assistant to facilitate the collection of an evidence-based medical history can result in more efficient teleconsultations, support asynchronous consultations, and support greater adherence to clinical protocols. However, we cannot conclude whether the use of the assistant increases the quality of the outcome of the consultation, and this needs to be further explored. Training for remote physicians and nurses on the limitations of TM and the indications for referral could improve the quality of care. TM has significant benefits, including improved health care access for women, reduced costs for the patient, and improved health system efficiency by reducing overcrowding at secondary and tertiary facilities and reducing the risk of COVID-19 transmission at facilities. Further research is needed to assess this approach's long-term outcomes, provider and patient satisfaction, and financial sustainability.

## Abbreviations

AB: Ayushman Bharat
ANC: antenatal care
ASHA: Accredited Social Health Activist
CHC: community health center
CHO: community health officer
F2F: face-to-face
HWC: health and wellness center
IRB: institutional review board
PHC: primary health center
TM: telemedicine

## Data Availability

The authors of this study have ensured that patient confidentiality is maintained and sensitive data were de-identified on the data sheets. This study’s data records and analyses are open for all curious researchers to explore and validate.

The authors of this study would like to share the full data sets underlying the results, available at [38], to ensure transparency in the results. This will enable other researchers to re-use the existing study protocols and build on a larger study in other geographies, allowing results to be independently verified.
